# Supplementary material for: Genomic Characterization of Aeromonas veronii Provides Insights into Taxonomic Assignment and Reveals Widespread Virulence and Resistance Genes throughout the World
Source: Antibiotics (Basel). 2023 Jun 11;12(6):1039. doi: 10.3390/antibiotics12061039 (PMC10295418; doi:10.3390/antibiotics12061039)
Supplement: Supplementary file 1 [file antibiotics-12-01039-s001.zip › antibiotics-2349969-supplementary.pdf]

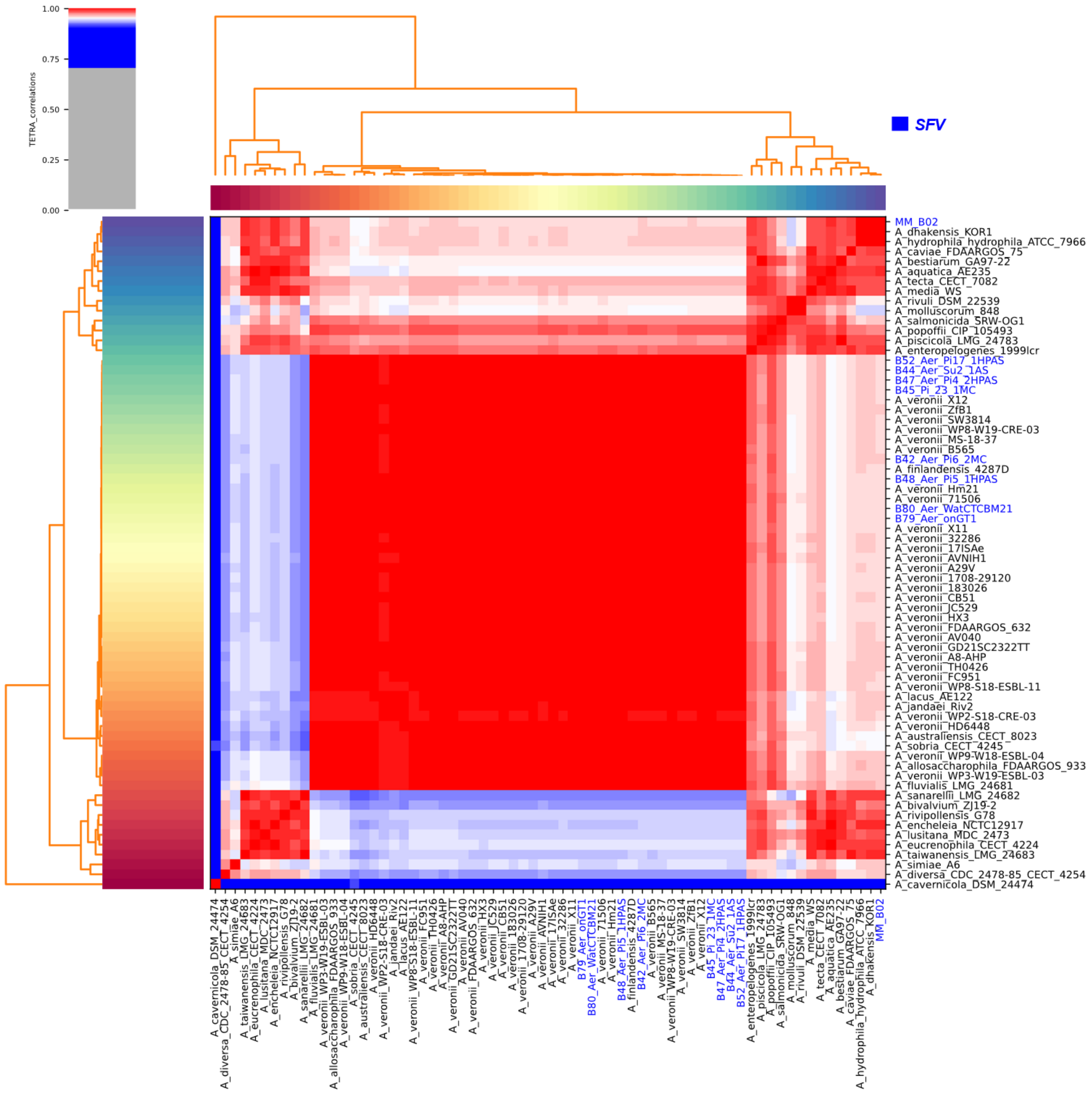

Figure S1: Heatmap illustrating the relationship between the genomes according to the pairwise calculated tetra nucleotide correlation index. San Francisco Valley (SFV) isolates highlighted in blue. Values closer to red indicate proximity to the maximum index (1,000). The figure was plotted as a standard output of the pyANI tool (Available online: https://github.com/widdowquinn/pyani, accessed on 21 May 2022).

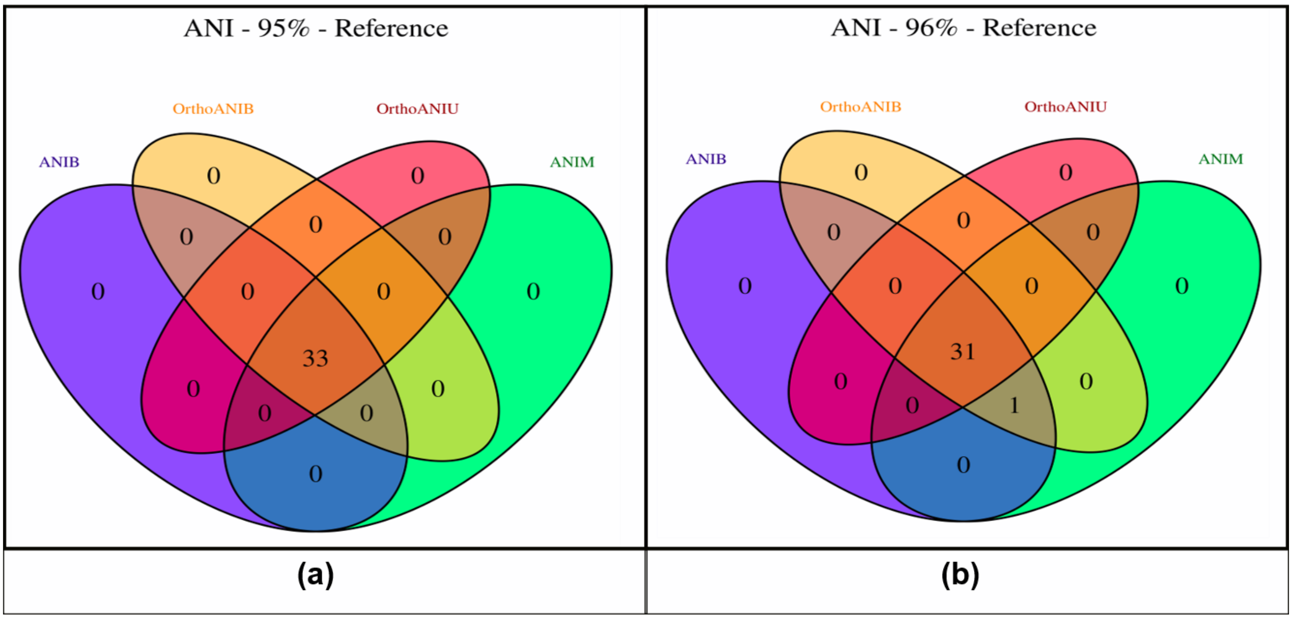

Figure S2: Venn diagrams illustrating the number of genomes ranked with the reference for the four methods for estimating average nucleotide identity according to the cutoff points tested: a) cut-off point equal to 95%; b) cut-off point equal to 96%. The figure was plotted with the R language (Available online: https://cran.r-project.org/, accessed on 3 January 2022) using the VennDiagram package (Available online: https://www.rdocumentation.org/packages/VennDiagram/versions/1.7.3/topics/venn.diagram, accessed on 23 August 2022).

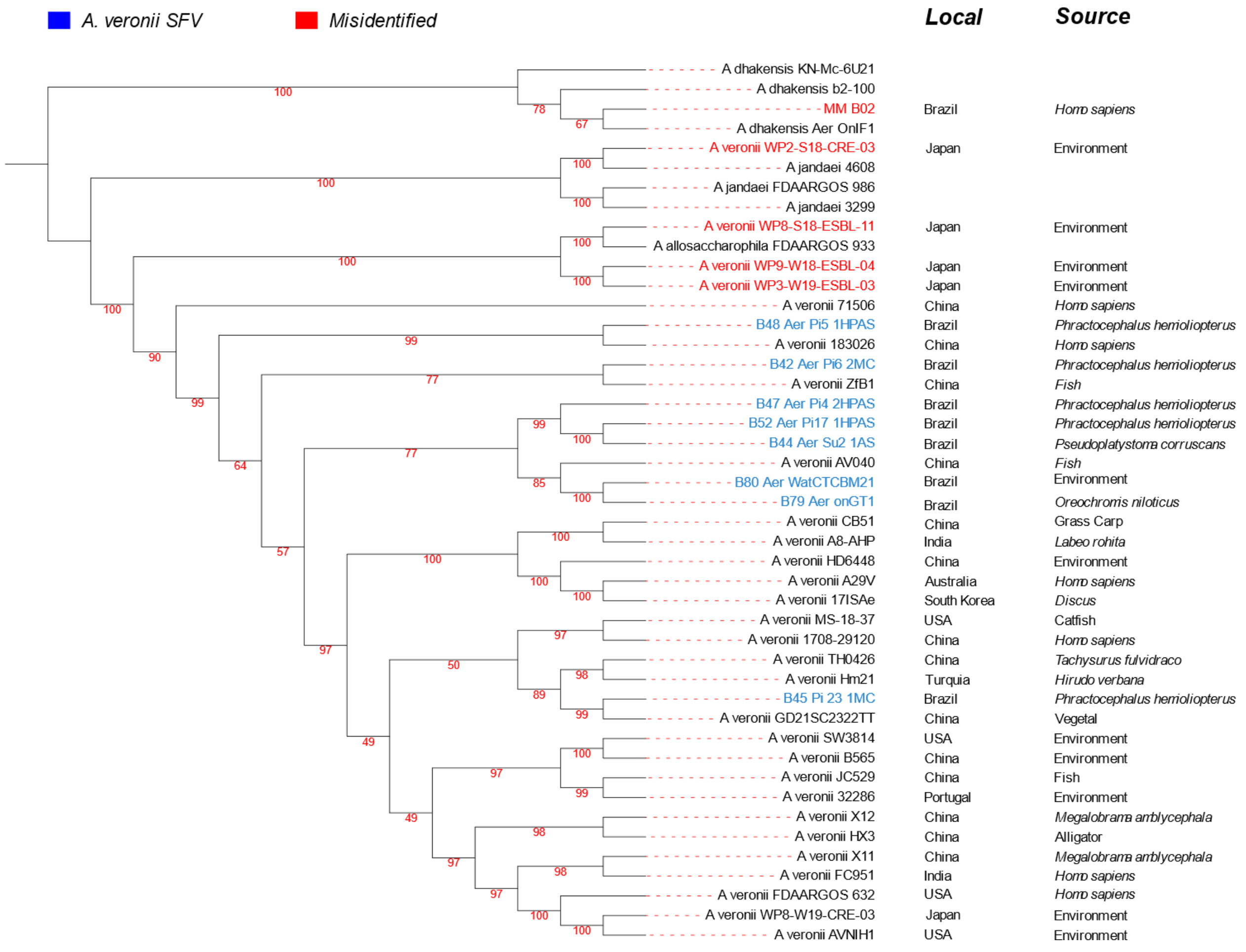

Figure S3: Phylogeny of SNPs from the core genome including bootstrap values (in red), sample location and isolation source. Misidentified isolates in red; SFV isolates of *A. veronii* in blue. The tree was edited using iTOL (Available online: https://itol.embl.de/, accessed on 15 August 2022) and host and isolation information was added using inkscape (Available online: https://inkscape.org/pt-br/, accessed on 14 October 2022).

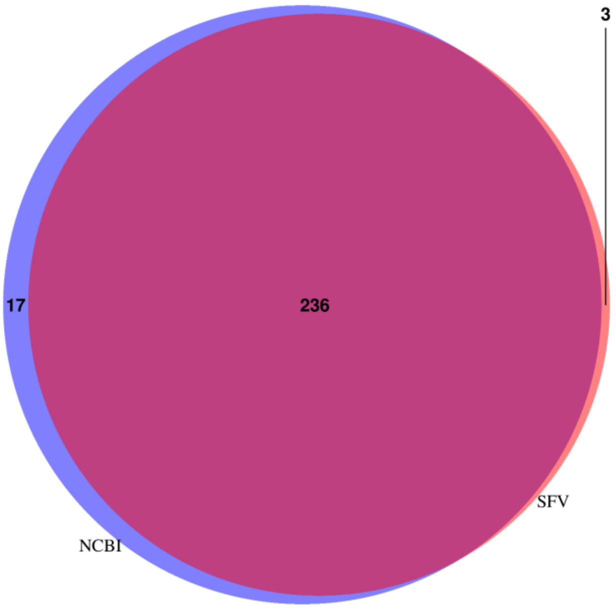

Figure S4: Venn Diagram illustrating the amount of virulence genes shared between SFV genomes and NCBI genomes, as well as genes particular to the two groups. The figure was plotted with the R language (Available online: https://cran.r-project.org/, accessed on 3 January 2022) using the VennDiagram package (Available online: https://www.rdocumentation.org/packages/VennDiagram/versions/1.7.3/topics/venn.diagram, accessed on 23 August 2022).

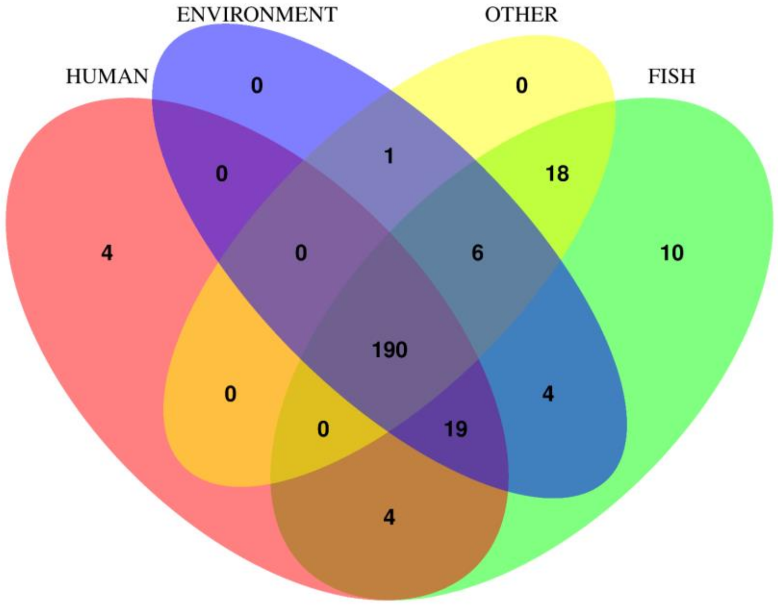

Figure S5: Veen Diagram illustrating the amount of genes shared between isolates from different isolation sources, as well as those that are unique to each. The figure was plotted with the R language (Available online: https://cran.r-project.org/, accessed on 3 January 2022) using the VennDiagram package (Available online: https://www.rdocumentation.org/packages/VennDiagram/versions/1.7.3/topics/venn.diagram, accessed on 23 August 2022).

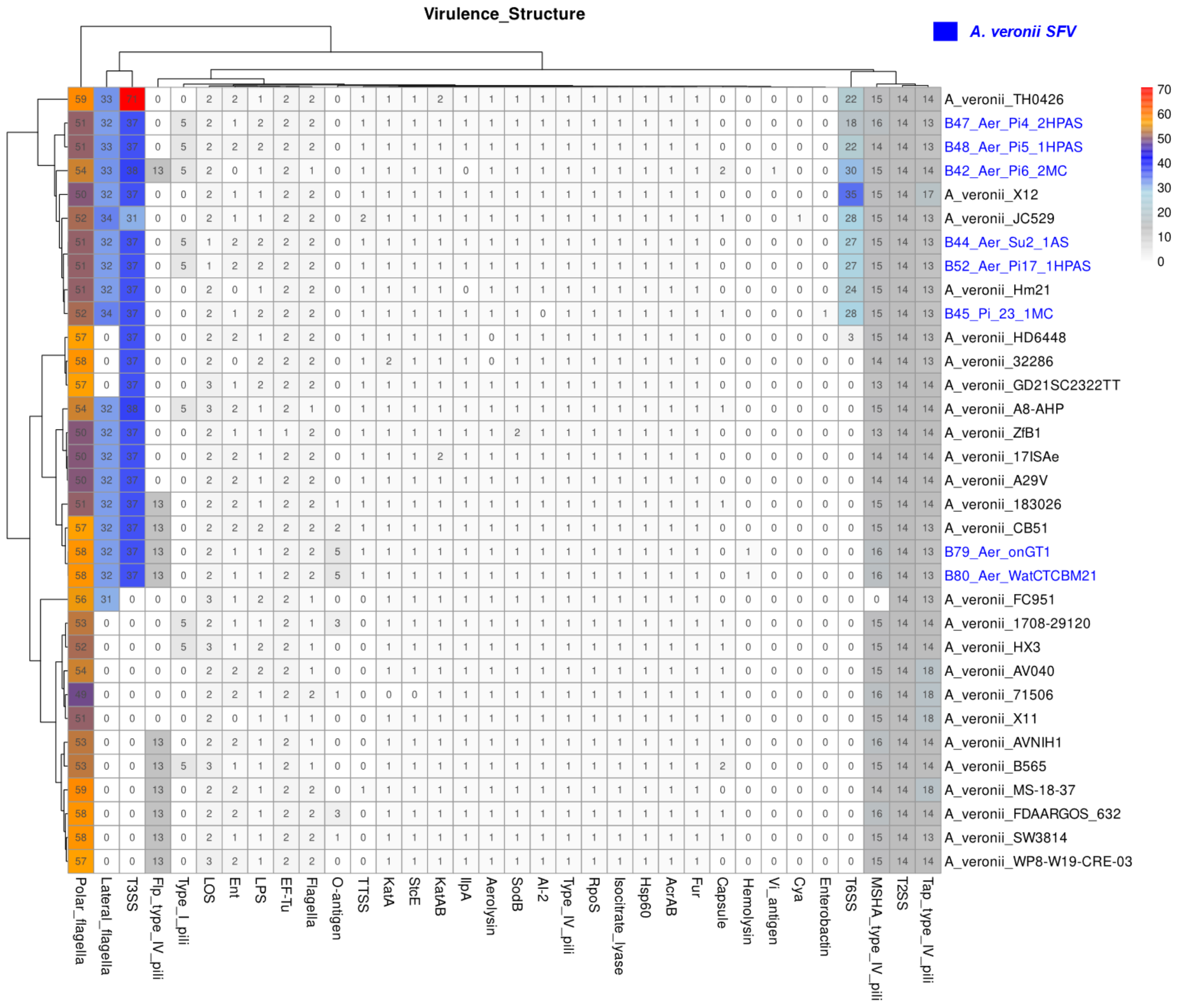

Figure S6: Amount of structure genes and virulence-related elements in each *Aeromonas veronii* isolate. SFV isolates highlighted in blue. The figure was plotted using R (Available online: https://cran.r-project.org/, accessed on 3 January 2022) with the package Pheatmap (Available online: https://www.rdocumentation.org/packages/pheatmap/versions/1.0.12/topics/pheatmap, accessed o 30 August 2022).

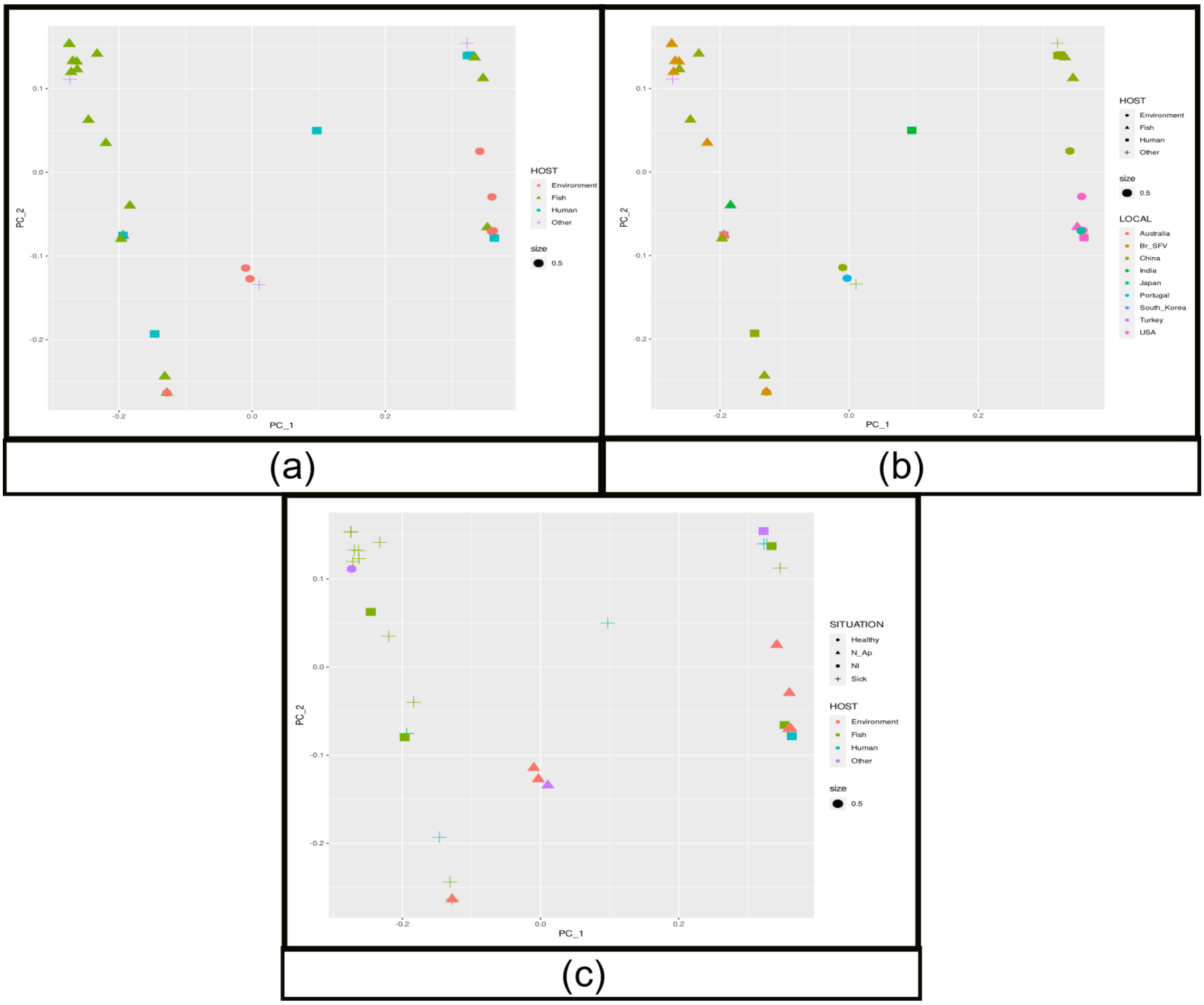

Figure S7: PCOA from presence/absence of genes plotted as scatter. a) Shape and color according to source of isolation. b) Shapes according to Host and color according to location. c) Shapes according to health status information and colors according to type of isolation source. The figure was plotted using R (Available online: https://cran.r-project.org/, accessed on 3 January 2022) with the package ggplot2 (Available online: https://ggplot2.tidyverse.org/, accessed on 4 January 2022).

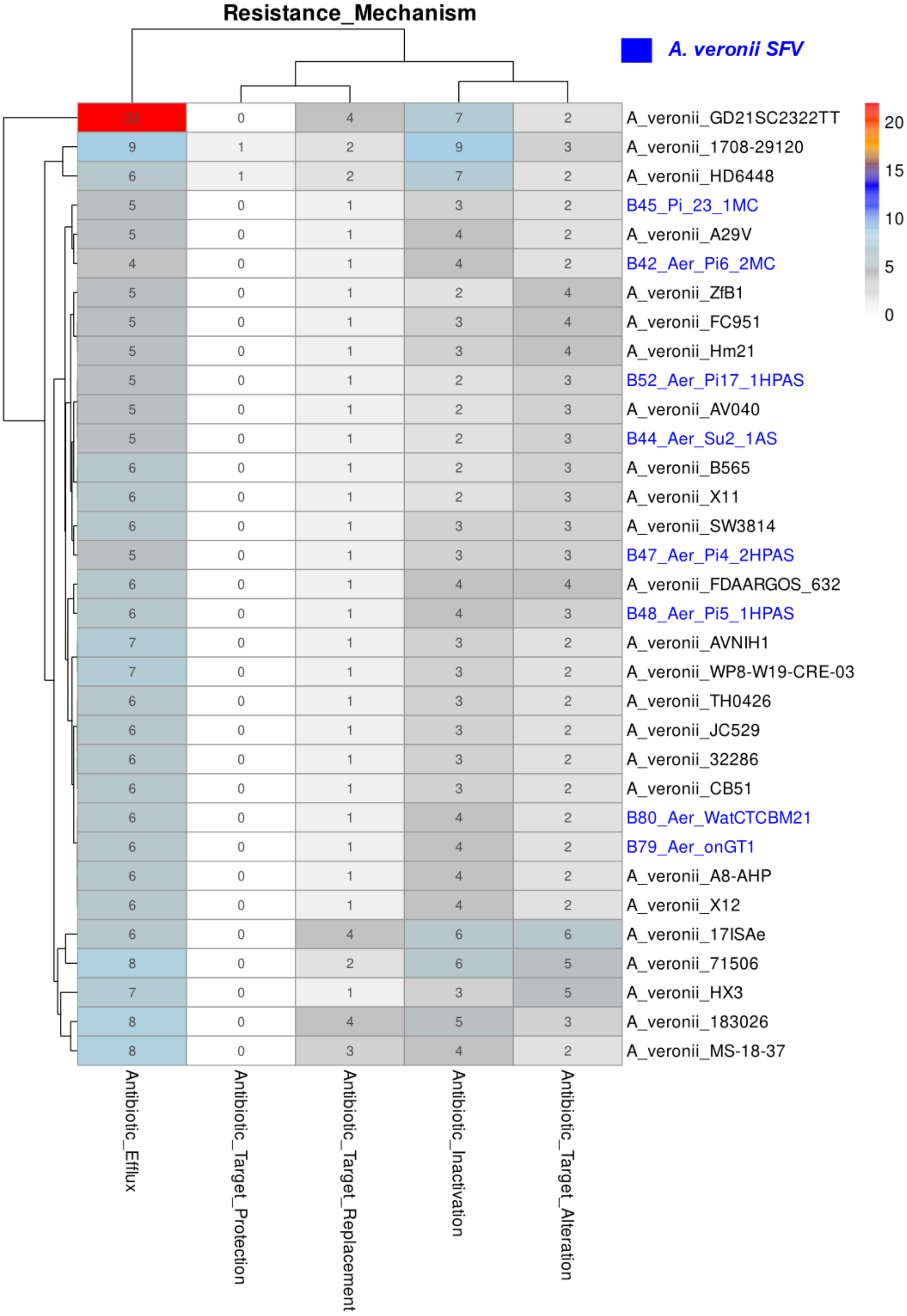

Figure S8: Amount of gene copies for each resistance mechanism in isolates. SFV isolates highlighted in blue. The figure was plotted using R (Available online: https://cran.r-project.org/, accessed on 3 January 2022) with the package Pheatmap (Available online: https://www.rdocumentation.org/packages/pheatmap/versions/1.0.12/topics/pheatmap, accessed o 30 August 2022).

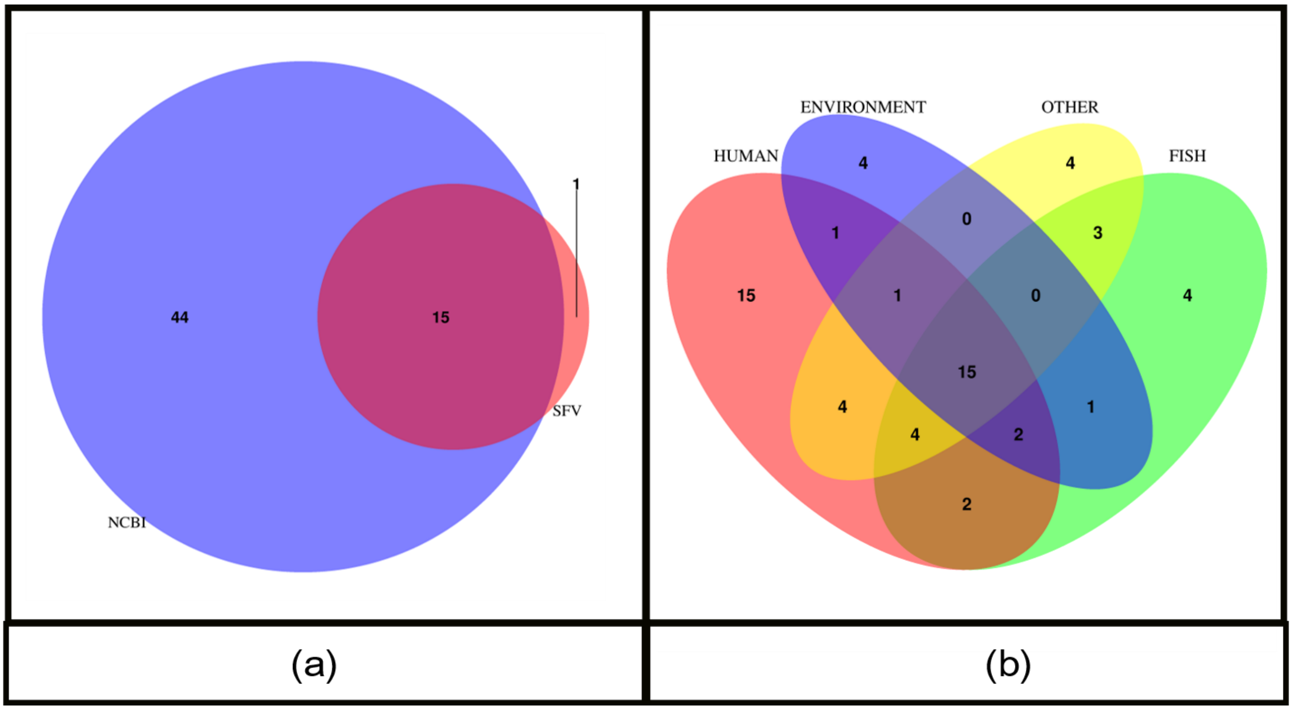

Figure S9: Venn Diagram illustrating the amount of antibiotic resistance-related genes shared between the SFV and NCBI isolates, as well as those that are unique to each group. The figure was plotted with the R language (Available online: https://cran.r-project.org/, accessed on 3 January 2022) using the VennDiagram package (Available online: https://www.rdocumentation.org/packages/VennDiagram/versions/1.7.3/topics/venn.diagram, accessed on 23 August 2022).

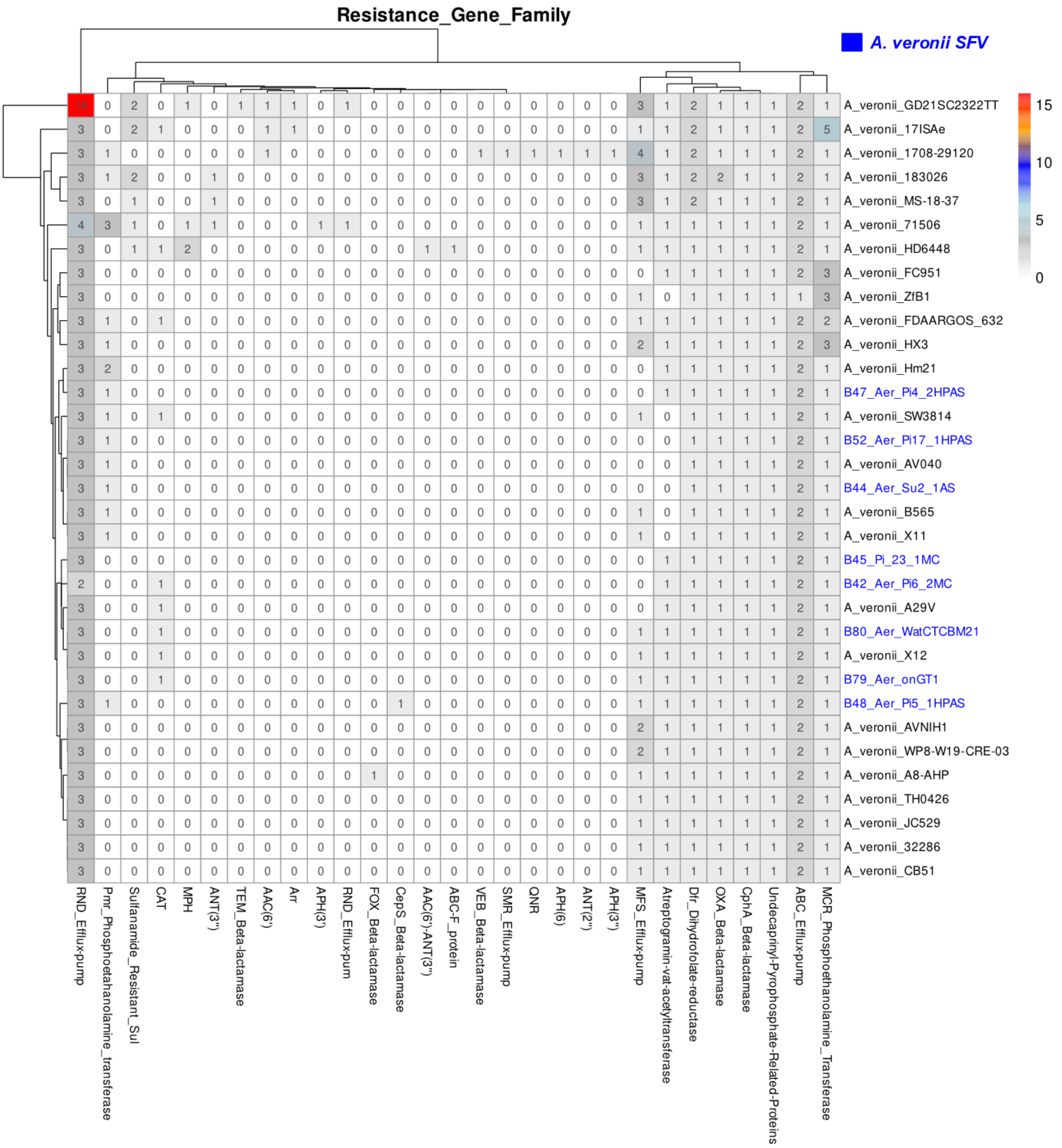

Figure S10: Figure illustrating the amount of genes from each gene family present in each genome. SFV isolates highlighted in blue. The figure was plotted using R (Available online: https://cran.r-project.org/, accessed on 3 January 2022) with the package Pheatmap (Available online: https://www.rdocumentation.org/packages/pheatmap/versions/1.0.12/topics/pheatmap, accessed o 30 August 2022).

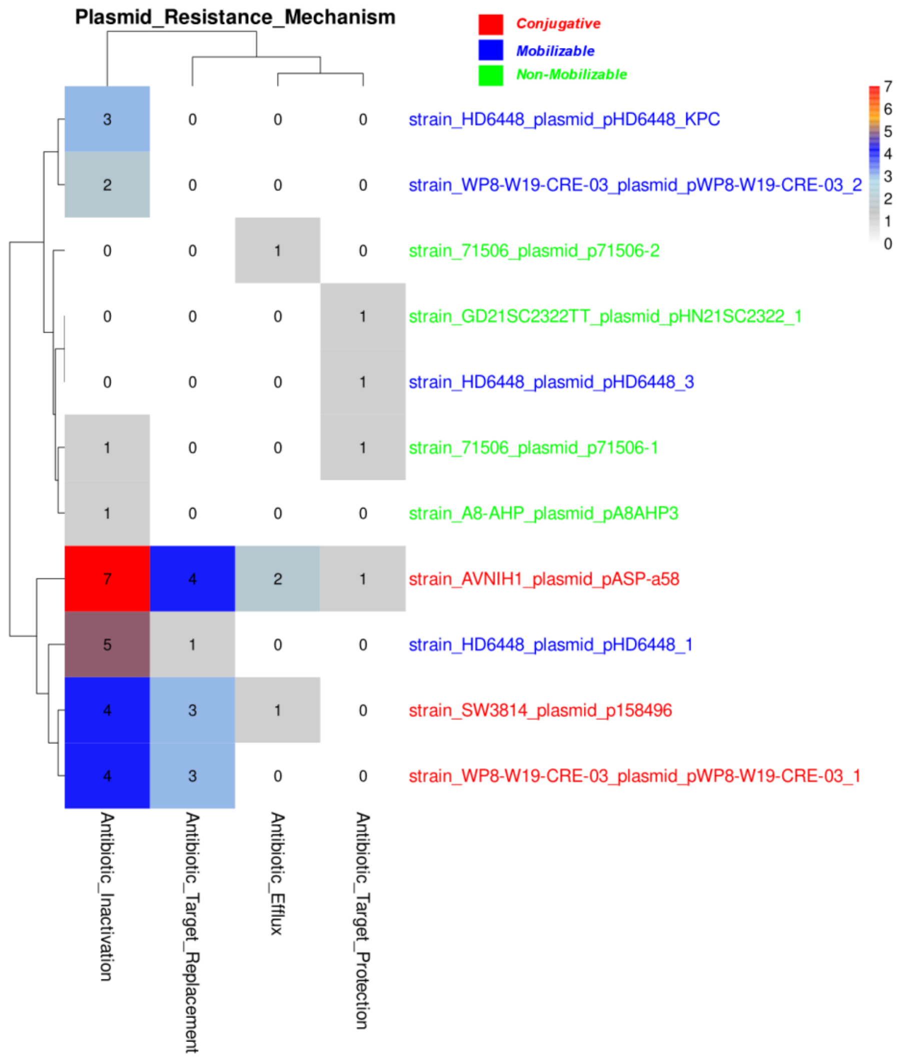

Figure S11: Number of genes present for each resistance mechanism in plasmids. Conjugative plasmids in red; mobilizable plasmids in blue; and unmobilizable plasmids in green. The figure was plotted using R (Available online: https://cran.r-project.org/, accessed on 3 January 2022) with the package Pheatmap (Available online: https://www.rdocumentation.org/packages/pheatmap/versions/1.0.12/topics/pheatmap, accessed o 30 August 2022).

Table S1: Result of mlst typing.

Genome	Genus	ST	Alele1	Alele2	Alele3	Alele4	Alele5	Alele6
1708-29120	Aeromonas	-	gyrB(~724)	groL(~225)	gltA(86)	metG(~748)	ppsA(~906)	recA(~840)
17ISAe	Aeromonas	485	gyrB(126)	groL(154)	gltA(354)	metG(16)	ppsA(353)	recA(374)
183026	Aeromonas	-	gyrB(~128)	groL(217)	gltA(143)	metG(679)	ppsA(~155)	recA(~146)
32286	Aeromonas	-	gyrB(~598)	groL(~591)	gltA(~802)	metG(785)	ppsA(686)	recA(~383)
71506	Aeromonas	-	gyrB(445)	groL(539)	gltA(453)	metG(~684)	ppsA(628)	recA(724)
A29V	Aeromonas	-	gyrB(126)	groL(154)	gltA(354)	metG(~766)	ppsA(~65)	recA(374)
A8-AHP	Aeromonas	-	gyrB(281)	groL(687)	gltA(~86)	metG(~226)	ppsA(~682)	recA(~805)
AV040	Aeromonas	-	gyrB(743)	groL(541)	gltA(302)	metG(~818)	ppsA(124)	recA(822)
AVNIH1	Aeromonas	333	gyrB(66)	groL(71)	gltA(255)	metG(175)	ppsA(124)	recA(255)
B565	Aeromonas	166	gyrB(19)	groL(18)	gltA(19)	metG(18)	ppsA(17)	recA(18)
CB51	Aeromonas	-	gyrB(599)	groL(556)	gltA(127)	metG(612)	ppsA(810)	recA(378)
FC951	Aeromonas	515	gyrB(377)	groL(313)	gltA(380)	metG(371)	ppsA(411)	recA(404)
FDAARGOS_632	Aeromonas	-	gyrB(66)	groL(71)	gltA(~350)	metG(~817)	ppsA(~511)	recA(223)
GD21SC2322TT	Aeromonas	-	gyrB(224)	groL(~73)	gltA(222)	metG(226)	ppsA(114)	recA(115)
HD6448	Aeromonas	1000	gyrB(724)	groL(457)	gltA(732)	metG(175)	ppsA(384)	recA(362)
Hm21	Aeromonas	-	gyrB(38)	groL(~661)	gltA(467)	metG(~677)	ppsA(511?)	recA(~799)
HX3	Aeromonas	-	gyrB(220)	groL(226)	gltA(802)	metG(~684)	ppsA(388)	recA(854)
JC529	Aeromonas	160	gyrB(136)	groL(141)	gltA(142)	metG(141)	ppsA(146)	recA(117)
MS-18-37	Aeromonas	254	gyrB(212)	groL(216)	gltA(151)	metG(213)	ppsA(84)	recA(220)
SW3814	Aeromonas	-	gyrB(130?)	groL(~700)	gltA(~203)	metG(~136)	ppsA(~133)	recA(~532)
TH0426	Aeromonas	-	gyrB(29)	groL(28)	gltA(17)	metG(840)	ppsA(26)	recA(28)
WP2-S18-CRE-03	Aeromonas	-	gyrB(~433)	groL(~423)	gltA(~639)	metG(~483)	ppsA(~724)	recA(823)
WP3-W19-ESBL-03	Aeromonas	-	gyrB(803)	groL(175)	gltA(54)	metG(~362)	ppsA(895)	recA(175)
WP8-S18-ESBL-11	Aeromonas	-	gyrB(~14)	groL(~740)	gltA(103)	metG(~317)	ppsA(~395)	recA(~871)
WP8-W19-CRE-03	Aeromonas	-	gyrB(66)	groL(71)	gltA(303)	metG(175)	ppsA(389)	recA(255)
WP9-W18-ESBL-04	Aeromonas	-	gyrB(100)	groL(~110)	gltA(173)	metG(172)	ppsA(~181)	recA(~840)
X11	Aeromonas	-	gyrB(479)	groL(221)	gltA(358)	metG(227)	ppsA(~859)	recA(53)
X12	Aeromonas	-	gyrB(466)	groL(~684)	gltA(56)	metG(~744)	ppsA(~869)	recA(~357)
ZfB1	Aeromonas	-	gyrB(407)	groL(480)	gltA(526)	metG(517)	ppsA(561)	recA(70)
B42_Aer_Pi6_2MC	Aeromonas	-	gyrB(~655)	groL(~702)	gltA(823?)	metG(~735)	ppsA(~655)	recA(~240)
B44_Aer_Su2_1AS	Aeromonas	-	gyrB(~813)	groL(~202)	gltA(613)	metG(~432)	ppsA(465)	recA(629)
B45_Pi_23_1MC	Aeromonas	-	gyrB(638)	groL(319)	gltA(32)	metG(129)	ppsA(651)	recA(~380)

B47_Aer_Pi4_2HPAS	Aeromonas	-	gyrB(~762)	groL(~202)	gltA(~225)	metG(416)	ppsA(72)	recA(~850)
B48_Aer_Pi5_1HPAS	Aeromonas	-	gyrB(~433)	groL(~591)	gltA(110)	metG(~586)	ppsA(~644)	recA(~240)
B52_Aer_Pi17_1HPAS	Aeromonas	-	gyrB(~813)	groL(~202)	gltA(613)	metG(~432)	ppsA(465)	recA(629)
B79_Aer_onGT1	Aeromonas	-	gyrB(553)	groL(~702)	gltA(319)	metG(~112)	ppsA(~229)	recA(~404)
B80_Aer_WatCTCBM21	Aeromonas	-	gyrB(553)	groL(~702)	gltA(319)	metG(~112)	ppsA(~229)	recA(~404)
MM_B02	Aeromonas	-	gyrB(376)	groL(302)	gltA(~812)	metG(504)	ppsA(100)	recA(~583)

ST: Sequence Type.

Table S2: *A. veronii* genomes downloaded from NCBI.

Genome	Host	Geral Location	Specific Location	Host Status	Plasmids
A8-AHP	*Labeo rohita* (Fish)	India	Punjab	Diseased	3
CB51	Grass Carp (Fish)	China	Hubei	Blood	0
WP9-W18-ESBL-04	WWTP (Environment)	Japan	Tóquio	Environment	4
X12	*Megalobrama amblycephala* (Fish)	China	Hubei	Cell Culture	0
AVNIH1	human-associated habitat (Environment)	USA	Bethesda	human-associated habitat	1
ZfB1	Fish	China	Hubei	-	0
GD21SC2322TT	Plants	China	Guangdong	Plants	1
SW3814	Lake Water (Environment)	USA	California	Environment	2
MS-18-37	Catfish (Fish)	USA	Mississipi	-	0
FC951	*Homo sapiens* (Human)	India	Vellore	Diarrhea	1
71506	*Homo sapiens* (Human)	China	Ningbo	Cholecystitis	2
17ISAe	*Discus* (Fish)	South Korea	-	Spleen	1
HX3	Alligator (Reptile)	China	-	-	1
183026	*Homo sapiens* (Human)	China	Hubei	Septicemia	0
WP8-W19-CRE-03	WWTP (Environment)	Japan	Tóquio	Environment	5
A29V	*Homo sapiens* (Human)	Australia	-	Gastroenteritis	2
AV040	Fish	China	Hefei	Liver-Spleen	0
1708-29120	*Homo sapiens* (Human)	China	Beijing	Cholangiolithiasis	0
X11	*Megalobrama amblycephala* (Fish)	China	Hubei	-	0
HD6448	Hospital Sewage (Environment)	China	Suzhou	Environment	5
32286	Waste Water (Environment)	Portugal	Lisboa	Environment	0
WP3-W19-ESBL-03	WWTP (Environment)	Japan	Tóquio	Effluent	5
TH0426	*Tachysurus fulvidraco* (Fish)	China	Zongguan	-	0
FDAARGOS_632	*Homo sapiens* (Human)	USA	-	-	1
WP8-S18-ESBL-11	WWTP (Environment)	Japan	Tóquio	Effluent	3
JC529	Carp (Fish)	China	Jilin	Sepsis	0
WP2-S18-CRE-03	WWTP (Environment)	Japan	Tóquio	Effluent	3
B565	Lake Sediment (Environment)	China	-	-	0
Hm21	*Hirudo verbana* (Annelid)	Türkiye	-	Healthy Digestive Tract	1

Table S3: Reference genomes of species of the genus *Aeromonas* downloaded from NCBI.

Species	Genome	Assembly
Aeromonas allosaccharophila	FDAARGOS_933	GCA_016026615.1
Aeromonas popoffii	CIP_105493	GCF_000820025.1
Aeromonas bestiarum	GA97-22	GCF_002906925.1
Aeromonas dhakensis	KOR1	GCA_001306015.1
Aeromonas bivalvium	ZJ19-2	GCA_003265465.1
Aeromonas fluvialis	LMG_24681	GCF_000819885.1
Aeromonas finlandensis	4287D	GCA_000764645.1
Aeromonas sobria	CECT_4245	GCA_000820145.1
Aeromonas eucrenophila	CECT_4224	GCF_000819865.1
Aeromonas salmonicida	SRW-OG1	GCF_012931585.1
Aeromonas enteropelogenes	1999lcr	GCA_000687355.2
Aeromonas piscicola	LMG_24783	GCA_000820005.1
Aeromonas aquatica	*AE235*	GCF_000764655.1
Aeromonas diversa	CDC_2478-85_CECT_4254	GCF_000819805.1
Aeromonas lacus	AE122	GCA_000764665.1
Aeromonas tecta	CECT_7082	GCA_000820185.2
Aeromonas cavernicola	DSM_24474	GCA_002795305.1
Aeromonas simiae	A6	GCA_014892695.1
Aeromonas caviae	FDAARGOS_75	GCF_000783715.2
Aeromonas media	WS	GCF_000287215.2
Aeromonas molluscorum	848	GCF_000388115.1
Aeromonas taiwanensis	LMG_24683	GCF_000699185.1
Aeromonas rivuli	DSM_22539	GCF_000820045.1
Aeromonas jandaei	Riv2	GCA_000708125.1
Aeromonas encheleia	NCTC12917	GCA_900637545.1
Aeromonas hydrophila hydrophila	ATCC_7966	GCF_000014805.1
Aeromonas australiensis	CECT_8023	GCF_000819725.1
Aeromonas rivipollensis	G78	GCF_010974825.1
Aeromonas sanarellii	LMG_24682	GCA_000820085.1
Aeromonas lusitana	MDC_2473	GCA_002812985.1

Table S4: Additional reference genomes included in the core genome phylogeny.

Species	Genome	Assembly
Aeromonas dhakensis	KN-Mc-6U21	GCA_002285935.1
Aeromonas dhakensis	b2-100	GCA_023920205.1
Aeromonas dhakensis	Aer_OnF1	GCA_022703095.1
Aeromonas jandaei	4608	GCF_018802325.1
Aeromonas jandaei	FDAARGOS 986	GCF_016127195.1
Aeromonas jandaei	3299	GCF_014217505.1
Aeromonas allosaccharophila	FDAARGOS_933*	GCA_016026615.1

*Same genome as Table S2.

Table S5: Putative plasmids identified in SFV (São Francisco Valley) isolates.

Genome	Plasmid	Mobility	Size (bp)	Circularized	mash_neighbor_identification
B44_Aer_Su2_1AS	AD562	Non-mobilizable	4831	Yes	*Aeromonas salmonicida*
B52_Aer_Pi17_1HPAS	AD562	Non-mobilizable	4831	Yes	*Aeromonas salmonicida*
B45_Pi_23_1MC	AD562	Non-mobilizable	4827	Yes	*Aeromonas salmonicida*
B45_Pi_23_1MC	AE566	Conjugative	144051	No	*Aeromonas hydrophila*

Table S6: Information on plasmids associated with isolates downloaded from NCBI.

PLASMIDS	STRAIN	MOBILITY	MASH_NEIGHBOR_IDENTIFICATION	RESISTANCE_GENES*
17ISAe_p_p17ISAe	17ISAe	non-mobilizable	*Aeromonas veronii*	-
71506_p_p71506-1	71506	non-mobilizable	*Aeromonas caviae*	2
71506_p_p71506-2	71506	non-mobilizable	*Aeromonas salmonicida subsp. salmonicida*	1
A29V_p_pAV1K	A29V	non-mobilizable	*Aeromonas caviae*	-
A29V_p_pAV7K	A29V	non-mobilizable	*Aeromonas veronii*	-
A8-AHP_p_pA8AHP1	A8-AHP	non-mobilizable	*Serratia marcescens*	-
A8-AHP_p_pA8AHP2	A8-AHP	non-mobilizable	*Aeromonas salmonicida*	-
A8-AHP_p_pA8AHP3	A8-AHP	mobilizable	*Mucilaginibacter sp. PAMC 26640*	1
AVNIH1_p_pASP-a58	AVNIH1	conjugative	*Aeromonas veronii*	14
FC951_p_unnamed	FC951	non-mobilizable	*Aeromonas veronii*	-
FDAARGOS_632_p_unnamed1	FDAARGOS_632	mobilizable	*Aeromonas veronii*	-
GD21SC2322TT_p_pHN21SC2322_1	GD21SC2322TT	non-mobilizable	*Aeromonas sp. ASNIH2*	1
HD6448_p_pHD6448_1	HD6448	mobilizable	*Aeromonas hydrophila subsp. hydrophila*	6
HD6448_p_pHD6448_3	HD6448	mobilizable	*Enterobacter kobei*	1
HD6448_p_pHD6448_4	HD6448	mobilizable	*Aeromonas salmonicida subsp. salmonicida*	-
HD6448_p_pHD6448_5	HD6448	mobilizable	*Aeromonas hydrophila*	-
HD6448_p_pHD6448_KPC	HD6448	mobilizable	*Pseudomonas aeruginosa*	3
Hm21_p_pHm21	Hm21	non-mobilizable	*Pantoea sp. PSNIH1*	-
HX3_p_pHX3	HX3	conjugative	*Aeromonas hydrophila*	-

SW3814_p_p158496	SW3814	conjugative	*Aeromonas hydrophila*	8
SW3814_p_p1739	SW3814	non-mobilizable	*Aeromonas caviae*	-
WP8-W19-CRE-03_p_pWP8-W19-CRE-03_1	WP8-W19-CRE-03	conjugative	*Aeromonas caviae*	7
WP8-W19-CRE-03_p_pWP8-W19-CRE-03_2	WP8-W19-CRE-03	mobilizable	*Aeromonas caviae*	2
WP8-W19-CRE-03_p_pWP8-W19-CRE-03_3	WP8-W19-CRE-03	mobilizable	-	-
WP8-W19-CRE-03_p_pWP8-W19-CRE-03_4	WP8-W19-CRE-03	mobilizable	*Aeromonas salmonicida subsp. salmonicida A449*	-
WP8-W19-CRE-03_p_pWP8-W19-CRE-03_5	WP8-W19-CRE-03	mobilizable	*Aeromonas sp. ASNIH2*	-

*Total amount.

Table S7: Assembly metrics of the genomes sequenced in this study.

Assembly	Size (pb)	N50	GC (%)	Contigs
B42_Aer_Pi6_2MC	4739098	323452	58.49	51
B79_Aer_onGT1	4576072	232736	58.75	72
B80_Aer_WatCTCBM21	4576144	253805	58.75	73
B48_Aer_Pi5_1HPAS	4705169	177529	58.73	72
B47_Aer_Pi4_2HPAS	4511905	249723	58.82	70
B52_Aer_Pi17_1HPAS	4559686	280995	58.62	73
B45_Pi_23_1MC	4630809	208383	58.65	58
B44_Aer_Su2_1AS	4559658	280995	58.62	76
MM_B02	4702809	299827	61.81	78
